# Sense and Nonsense of an Extended Pelvic Lymph Node Dissection in Prostate Cancer

**DOI:** 10.1155/2012/983058

**Published:** 2011-10-09

**Authors:** Anthony Van Baelen, Nicolas Mottet, Martin Spahn, Alberto Briganti, Paolo Gontero, Steven Joniau

**Affiliations:** ^1^Department of Urology, University Hospitals Leuven, 3000 Leuven, Belgium; ^2^Department of Urology, Clinique Mutualiste de la Loire, 42013 Saint-Etienne, France; ^3^Department of Urology, University of Würzburg, 97070 Würzburg, Germany; ^4^Department of Urology, Vita-Salute University, 20132 Milan, Italy; ^5^Department of Urology, University of Turin, 10129 Turin, Italy

## Abstract

Lymph node metastases associated with prostate cancer (PCa) has been shown to be a poor prognostic factor. The role of pelvic lymph node dissection (PLND) itself in relation to survival remains unclear, however. A Medline search was conducted to address this issue. The following conclusions were drawn. Only recently, improved survival due to completion of radical prostatectomy (RP) (compared to abandoning RP) in known or presumed lymph-node-positive patients has been shown. Lymph node sampling can only be considered representative if an adequate number of nodes is removed. While several authors have suggested that a therapeutic benefit in patients undergoing RP is not provided by PLND, the reliability of these studies is uncertain. Contrary to this, several studies have indicated the possibility of long-term survival even in the presence of limited lymph node metastases. The role and timing of initiation of adjuvant androgen deprivation therapy (ADT) in patients who have node-positive disease after RP is controversial. Recent studies suggest that delaying ADT may not adversely impact survival.

## 1. Introduction

Lymph node metastases in men diagnosed with PCa has been shown to be a poor prognostic factor for biochemical recurrence (BCR) and survival [1–3]. As part of the stage migration in PCa during the PSA era, the incidence of positive lymph nodes has decreased steadily [1, 4]. Nevertheless, accurate identification and staging of men with lymph node metastases allows more precise prognostication and may have important implications on the initiation of adjuvant therapy [5]. Surgical excision and histological examination of the pelvic lymph nodes provides the most accurate staging information regarding pelvic lymph node status [6–8]. The role of PLND in relation to survival remains, however, unclear. Furthermore, switching from a limited nodal dissection to an extended one (ePNLD) is associated with a 14.6% complications rate which in most papers is higher than that of a more limited dissection [8]. Of course, the benefit of performing an ePLND must outweigh the elevated risk of complications.

We sought to review the available literature concerning the role of PLND and RP on survival outcome.

## 2. Evidence Acquisition

A Medline search was conducted to identify original articles addressing the role of PLND in PCa. Keywords included prostate cancer, pelvic lymph node dissection, and radical prostatectomy. All of the keywords are within the Medical Subject Headings (MeSH) database. Original articles with the highest level of evidence were identified and were critically reviewed.

## 3. Evidence Synthesis

### 3.1. Localization of Nodes Draining the Prostate

The lymphatic drainage of the prostate gland has been described more than 100 years ago [9]. Three regions are concerned: common iliac, external iliac, and hypogastric. These anatomic findings have been confirmed clinically through extended nodal dissections revealing positive nodes outside the obturator fossa, namely, around the common iliac artery and the hypogastric pedicle in 22% and 29%, respectively [10]. Therefore, all these sites have to be considered while performing an extended pelvic lymph node dissection.

### 3.2. Number of Nodes Removed

Since the number of positive lymph nodes detected is strongly correlated to the number of nodes removed, lymph node sampling can only be considered representative if an adequate number of nodes and all relevantly located nodes are removed [4–8, 11–16]. An autopsy study by Weingärtner et al. suggests that 20 lymph nodes must be removed for an adequate PLND [17]. Several studies have shown this requirement to be technically feasible by harvesting a comparable number of lymph nodes in vivo [2, 7, 8, 13, 18, 19]. Reports on patients who underwent more limited PLND might be misleading and incomplete lymph node sampling probably will not provide an accurate prognostic factor for survival. A certain percentage of cases would be considered lymph node negative (pN0) because metastases were not detected merely by the limited extent of PLND performed. Such patients would be left with residual tumor and perhaps deprived of the chance of cure.

However, maybe being even more important than the absolute number of nodes, the anatomical boundaries of PLND should be considered since there is a natural variability in the number of nodes encountered in one patient compared to another. Moreover, as is the case in radical cystectomy, the differences in methods by which lymphatic tissues are collected, transported, and processed by the pathologist could lead to a significant difference in nodal count. Thus, the absolute number of nodes counted might be misleading [20]. 

Irrespective of the nodal status, an increased absolute number of nodes removed have been suggested to be associated with better cancer control for some tumors, such as colon or bladder cancer [21]. In PCa, this has been tested in only one retrospective cohort, without any benefit for relapse [14]. But major limitations must be highlighted, such as the median number of removed nodes (only 9), and a statistical analysis performed on only 174 patients with positive nodes. Using the SEER database, a positive relationship was observed between the total number of nodes removed, and PCa-specific survival. Surprisingly, the difference was observed in PLND with >4 nodes, which is far below the expected number of an ePLND [13, 16]. Confirmatory analysis based on prospective data is mandatory.

### 3.3. Indications for PLND

Indications for PLND are subject to debate. The guidelines of the European Association of Urology indicate that a PLND should be undertaken in men with intermediate (cT2a, PSA 10–20 ng/mL, biopsy Gleason score 7) or high-risk (>cT2b, PSA >20 ng/mL, Gleason score ≥8) PCa, when the nomogram-estimated risk for positive nodes exceeds 7% [22]. Meanwhile, there are other authors who recommend performing ePLND in all patients with a PSA level of ≥10 ng/mL, and in all patients with a PSA level of <10 ng/mL with a Gleason score >6 [4]. The definition of the indication for PLND lies not within the scope of this paper, nor is the incorporation of sentinel node sampling within the diagnostic armamentarium.

### 3.4. pN0 Patients

Several authors have suggested that a therapeutic benefit in patients undergoing RP is not provided by PLND. DiMarco et al. reported that the extent of lymphadenectomy does not affect cancer outcome in lymph node negative cases thereby making the impact of PLND negligible in the overall population, or in patients stratified as having high-risk PCa based on the D'Amico classification (1059 high risk patients) [23]. This single institution analysis on a cohort of 7036 patients undergoing RP with or without PLND over a 13 yr time span has several limitations. Since conclusions were drawn from a pN0 PCa population of which 71% of patients had Gleason score ≤6, 95% had organ confined disease and only 26% had a PSA >10 ng/mL, no definite conclusions can be drawn for the entire PCa population, especially for patients with high risk PCa or lymph node invasion (pN1). Moreover, the mean number of nodes removed per patient in this study was only 9. Bearing in mind the strong correlation between the total number of nodes removed and the number of positive nodes detected, underestimation of lymph node invasion is inherent to more limited PLND rendering any conclusion on survival based on a less than extended PLND doubtful. Finally, therapeutic outcome in terms of BCR was the same between the patients operated at the beginning of the author's experience with a more extensive PLND compared to those operated 10 yr later with a minimal PLND despite a significant shift in tumor stage. It cannot be excluded that more extensive PLND in their early experience might have had an influence on recurrence and survival because one would expect a poorer outcome for the earlier group, considering the higher frequency of locally advanced PCA in this era.

Murphy et al. showed in a similar population of 964 pN0 patients with low median PSA of 6.2 ng/mL, of which only 36.5% of patients had Gleason score ≥7 and 99.5% had clinically organ confined disease, that the number of nodes removed was not significantly associated with BCR [24], thereby questioning the role of PLND. Again this type of population does not warrant conclusions for PCa patients at higher risk of disseminated disease. Moreover, the anatomical extent of the PLND performed was not captured in this study. The internal iliac region is often omitted when performing PNLD, although lymph node metastasis is said to be found in this region in 62% of cases [13]. The absence of knowledge on the regional extent of PLND might introduce an inherent bias.

Bhatta-Dhar et al. also failed to find a statistically significant difference in the 6-year BCR-free survival rate in a low-risk PCa population who did or did not undergo PLND [25]. Conclusions for the entire population of PCa patients (with intermediate and high-risk patients included) cannot be drawn, especially not for those with pN1 PCa. Moreover, a followup of 6 years is too short when considering the typical protracted natural history of PCa.

In summary, the reliability of studies showing that PLND does not confer a survival benefit in pN0 is uncertain. 

A last comment regards the definition of pN0 patients. Are the analyzed nodes really negative? In a retrospective single centre analysis of 4,611 patients, Masterson et al. showed a positive impact on BCR-free survival resulting from an increased total number of nodes removed in pN0 patients [14]. It was speculated that this might have been due to cellular disease that had escaped identification, but was treated effectively with ePLND. This may indeed be considered since using modified pathological analysis such as systematic immunohistochemistry analysis [26] or RT-PCR [27] increases the node positivity by 13.3% and 26.6%, respectively. Interestingly, patients with an upstaged nodal status using those techniques showed the same cancer-related outcomes compared to those who were initially staged pN1.

### 3.5. pN1 Patients

Although no prospective randomized clinical trials are available, several studies have indicated the possibility of long-term survival even in the presence of limited lymph node metastases [3, 5, 6, 16, 18].

Based on a population of 367 patients who underwent RP and extended PLND, Bader et al. concluded that some patients with minimal metastatic disease in the lymph nodes remain free of BCR for more than 10 years after surgery without adjuvant treatment [18]. This finding implies a possible therapeutic effect of PLND, especially since in this report any adjuvant treatment was deferred until symptoms of disease progression occurred.

Likewise, Allaf et al. showed that a significant benefit in BCR-free survival may exist for certain subgroups of pN1 PCa patients undergoing RP with extended PLND [5]. In this single centre study including 4,000 patients, the outcomes of RP with limited PLND (all performed by surgeon A) were compared to those of RP with extended PLND (performed by surgeon B). There was a trend towards improved BCR-free survival in patients who underwent extended dissection. In a subset of patients with nodal involvement and less than 15% positive lymph nodes, there was a statistically significant difference between PLND techniques with respect to BCR. However, given the low number of patients with positive lymph nodes, this study could be underpowered.

The same group retrospectively analyzed a population of 3,264 patients undergoing RP with extended PLND [28]. Nodal involvement was present in 143 patients (4.4%), and these patients only underwent adjuvant treatment after clinical disease recurrence occurred. In the multivariate analysis, 15% or greater positive lymph nodes was a significant predictor of BCR. Stratifying patients simultaneously according to the 3 strongest prognostic factors made it possible to define a subset of pN1 patients that showed 5-year BCR-free survival of 52% (i.e., for patients with <15% positive lymph nodes and Gleason score ≤7 and absence of seminal vesicle invasion). An important limitation of this study was that BCR was defined as end-point, rather than clinical progression or survival. 

The concept and prognostic significance of the percentage of positive lymph nodes or lymph node density was further elaborated in a single centre retrospective analysis of 235 pN1 patients after RP with extended PLND [2]. 69% of these patients did not undergo any adjuvant treatment. Lymph node density was defined as the number of positive nodes divided by the total number of nodes removed. When stratified by lymph node density, patients with a density of 20% or greater were at higher risk for clinical recurrence compared to those with a density of less than 20% (RR: 2.31; *P* < 0.001). In patients with lymph node density less than 20% the mean 10-year clinical recurrence-free survival rate was 72% compared to 47% in those with a lymph node density 20% or greater.

Probability of long-term survival after RP with or without subsequent androgen deprivation therapy, even with the presence of limited lymph node metastases, was also observed in a study of 13,020 patients undergoing RP obtained from the Surveillance, Epidemiology, and End Result Program (SEER) [16]. Patients undergoing excision of at least 4 lymph nodes (node-positive and node-negative patients) or more than 10 nodes (only node-negative patients) had a lower risk of prostate cancer-specific death at 10 years than did those who did not undergo PLND. This is only possible if a percentage of patients with macroscopic as well as microscopic lymph node invasion is cured by more extensive PLND. Using the SEER database poses several evident limitations: lack of information regarding adjuvant hormonal treatment, lack of information on other patient characteristics, inability to control for margin status, and lack of PSA data for some of the patients included.

A large retrospective analysis from the Mayo Clinic confirmed the finding that RP may offer long-term survival to patients with pN1 PCa [11]. In 507 patients identified as pN1 at RP with extended PLND, cancer-specific survival (CSS) depended amongst other variables on the degree of lymph node involvement. Ten-year CSS was as high as 86%. While in this last study 90% of patients received adjuvant ADT, Schumacher et al. reported on 122 pN1 patients after RP who did not receive any ADT. They confirmed good long-term BCR-free survival and CSS for patients with low-volume nodal burden after RP [6]. For patients with 1 positive lymph node, 10-year BCR-free survival and CSS were 24.7% and 72.1%, respectively. In patients with 2 positive lymph nodes, 10-year BCR-free survival and CSS were still 11.8% and 79.1%, respectively.

The exact definition of “low-volume nodal burden” portending good survival was further elaborated by Fleischmann et al. in a single-centre study of 102 node-positive patients who did not receive adjuvant ADT [15]. On multivariate analysis, the diameter of the largest metastasis was the strongest prognostic factor for all end points (BCR, CSS, and overall survival). On the other hand, Briganti et al. emphasize on the absolute number of nodes affected when they state that ≥2 positive nodes represent a significant cutoff value for predicting CSS in patients with node-positive PCa after RP [7, 8]. Important limitations of this study were the fact that all patients were submitted to adjuvant ADT, differences in population characteristics between the two contributing institutions as well as differences in the way the lymph nodes were sent for pathological examination.

### 3.6. cN1 Patients

Until recently, there was only limited data to support completing RP in known or presumed lymph-node-positive patients. Using a population-based database, Engel and Bastian showed that patients with pN1 disease at frozen section analysis may experience improved survival when RP is completed, compared to patients with abandoned RP [29]. This obvious advantage should encourage urologists to complete RP, regardless of lymph node status, and therefore omit frozen section analysis of lymph nodes. Their conclusions were based on 13,805 patients with histologically confirmed PCa included in the Munich Cancer Registry. Differences in survival were impressive, and in favor of patients with completed RP, even in node-positive cases. Of course, a formal prospective trial would be the only way to get a definitive answer on this issue, but this is unlikely to ever be done.

### 3.7. Adjuvant Treatments

Although ADT has a well-defined role in patients who have metastatic disease and in patients who are undergoing radiotherapy for high-risk disease, its role in patients who have node-positive disease after RP is controversial. Messing et al. reported the results of a randomized, controlled clinical trial in men with pN1 after RP, comparing life-long immediate adjuvant ADT with ADT at the time of metastatic disease. The study reported a significant advantage in progression-free survival and overall survival that favored early adjuvant ADT [30]. Given the potential for long-term adverse effects associated with ADT, such as osteoporosis, cardiovascular disease, diabetes, and mood disorders, delaying the initiation of ADT until documented BCR may spare patients significant treatment-related toxicities. In a large cohort of 731 pN1 patients after RP, Wong et al. compared administration of adjuvant ADT with patients who did not receive adjuvant ADT. The results suggest that the delay of ADT may not adversely impact CSS, nor overall survival [31]. This cohort of patients was constructed using linked SEER—Medicare data implying several limitations as already has been mentioned before. In a similar setting, Schröder et al. reported on the result of EORTC 30846, which examined the role of immediate versus delayed ADT in patients who had node-positive disease and who did not undergo RP. There was no difference in overall survival between the early and deferred ADT arms [32]. The most likely reason for these conflicting results is the difference in indication for initiation of ADT in the groups that were initially observed. In the study by Messing et al., patients were only started on ADT if they developed clinical metastases, which is associated with a high risk of both CSS and overall mortality. However, in more recent trials, post-RP monitoring of PSA became routine and patients in these series likely were followed for BCR. Hypothetically, treatment with ADT at the time of BCR may be successful in the treatment of micrometastatic disease and in the prevention of the onset of metastatic disease (and possibly subsequent death from PCa). This hypothesis may be a possible explanation for the improved outcomes in the delayed treatment arm of the above-mentioned series [31, 32]. Further studies support such hypothesis. D'Amico et al. proved a PSA doubling time of less than 3 months is a surrogate end point of CSS. Therefore, these men are most likely to benefit most from the extended, relatively symptom-free interval provided by early salvage ADT [33]. Whether or not adding ADT at the time of BCR actually improves survival remains to be clarified. In the literature, only few retrospective papers show evidence of some improvement in CSS and even in overall survival [34, 35]. Anyhow, the promising survival rate of patients with very low-volume nodal burden after surgery alone (RP and PLND) makes the value of routine immediate ADT in all patients with lymph node metastases at least questionable, especially when considering the negative side effects [18]. 

Another interesting aspect of adjuvant therapy was alluded to by da Pozzo et al. [19]. This group was the first to investigate the role of ADT with or without radiation therapy in node-positive patients. All 250 patients underwent RP with extended PLND and were submitted to adjuvant ADT in this single centre report. They found a significant protective role for adjuvant ADT together with radiation therapy in CSS. Important limitations of this study are the two different radiation therapy regimens that were used (26% underwent irradiation of the prostatic bed only, while 74% also underwent pelvis irradiation) and data regarding quality of life related to the delivery of adjuvant radiation therapy were not obtained. Therefore, it can so far only be considered as hypothesis generating and cannot be considered standard of care.

## 4. Conclusions

From this Medline search concerning the role of PLND and RP on survival outcome, the following conclusions were drawn. First, lymph node sampling can only be considered representative if an adequate number of nodes and all relevantly located nodes are removed. Second, while several authors have suggested that a therapeutic benefit in patients undergoing RP is not provided by PLND, the reliability of these studies is uncertain. Third, several studies have indicated the possibility of long-term survival even in the presence of very limited lymph node metastases provided patients had an extended PLND. Fourth, improved survival after completion of RP (compared to abandoning RP) in known or presumed lymph-node-positive patients has recently been shown. Fifth, although ADT has a well-defined role in patients who have metastatic disease or in patients with high-risk localized disease undergoing radiotherapy, its role in patients who have node-positive disease after RP is controversial. Historically, a significant advantage in progression-free survival and overall survival was attributed to immediate adjuvant ADT after RP. However, this evidence was acquired in a pre-PSA, small-sized prospective randomized trial in which most patients had high-volume nodal tumor burden. The translation of those results towards contemporary pN1 patients—who frequently have low-volume nodal tumor burden in an extended PLND—is problematic.
